# Supplementary material for: A tailored tetravalent peptide displays dual functions to inhibit amyloid β production and aggregation
Source: Commun Biol. 2023 Apr 8;6:383. doi: 10.1038/s42003-023-04771-9 (PMC10082830; doi:10.1038/s42003-023-04771-9)
Supplement: Supplementary file 2 — Supplementary Figures [file 42003_2023_4771_MOESM2_ESM.pdf]

A tailored tetravalent peptide displays dual functions to inhibit amyloid β production and aggregation

W. Sato et al.

Supplementary Figs. 1-12

1st screening
1 2 3 4 5 6 7
XXXXXXX-U

Supplementary Fig. 1a

	1	2	3	4	5	6	7	8	9	10	11	12	13	14	15	16	17	18	19	20	21	22	23	24
A		1A	2A	3A	4A	5A	6A	7A	1R	2R	3R	4R	5R	6R	7R	1N	2N	3N	4N	5N	6N	7N	1D	2D
B	3D	4D	5D	6D	7D	1Q	2Q	3Q	4Q	5Q	6Q	7Q	1E	2E	3E	4E	5E	6E	7E	1G	2G	3G	4G	5G
C	6G	7G	1H	2H	3H	4H	5H	6H	7H	1I	2I	3I	4I	5I	6I	7I	1L	2L	3L	4L	5L	6L	7L	1K
D	2K	3K	4K	5K	6K	7K	1M	2M	3M	4M	5M	6M	7M	1F	2F	3F	4F	5F	6F	7F	1P	2P	3P	4P
E	5P	6P	7P	1S	2S	3S	4S	5S	6S	7S	1T	2T	3T	4T	5T	6T	7T	1W	2W	3W	4W	5W	6W	7W
F	1Y	2Y	3Y	4Y	5Y	6Y	7Y	1V	2V	3V	4V	5V	6V	7V										

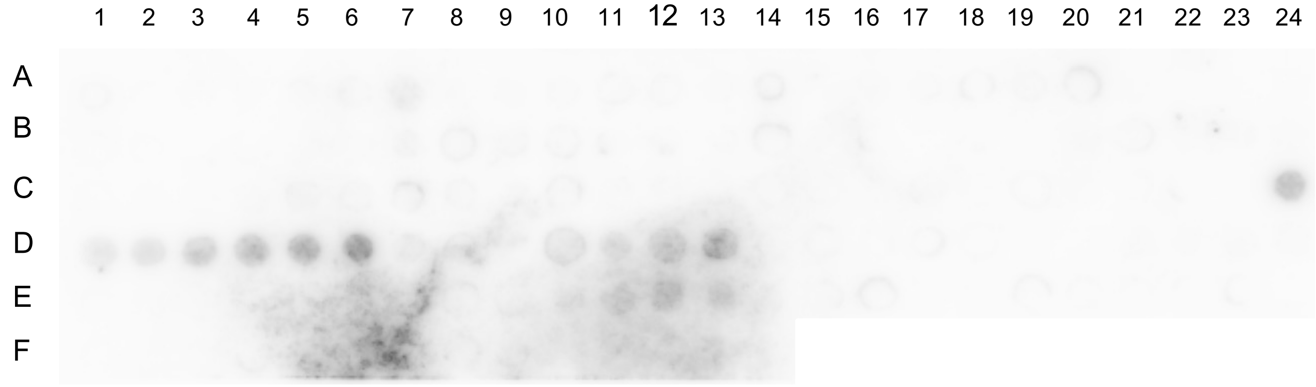

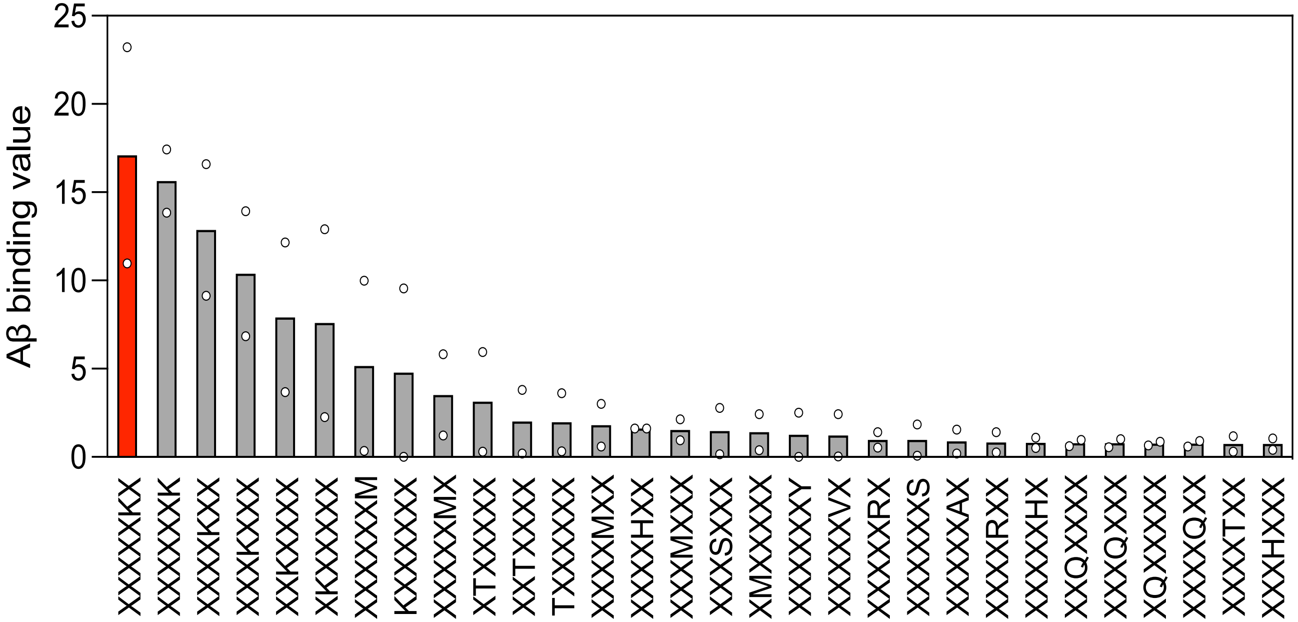

Supplementary Fig. 1b

2nd screening
1 2 3 4 5   7
XXXXXKX-U

	1	2	3	4	5	6	7	8	9	10	11	12	13	14	15	16	17	18	19	20	21	22	23	24
A		1A	2A	3A	4A	5A	7A	1R	2R	3R	4R	5R	7R	1N	2N	3N	4N	5N	7N	1D	2D	3D	4D	5D
B	7D	1Q	2Q	3Q	4Q	5Q	7Q	1E	2E	3E	4E	5E	7E	1G	2G	3G	4G	5G	7G	1H	2H	3H	4H	5H
C	7H	1I	2I	3I	4I	5I	7I	1L	2L	3L	4L	5L	7L	1K	2K	3K	4K	5K	7K	1M	2M	3M	4M	5M
D	7M	1F	2F	3F	4F	5F	7F	1P	2P	3P	4P	5P	7P	1S	2S	3S	4S	5S	7S	1T	2T	3T	4T	5T
E	7T	1W	2W	3W	4W	5W	7W	1Y	2Y	3Y	4Y	5Y	7Y	1V	2V	3V	4V	5V	7V				ori	

ori: original motif; XXXXXKX-U

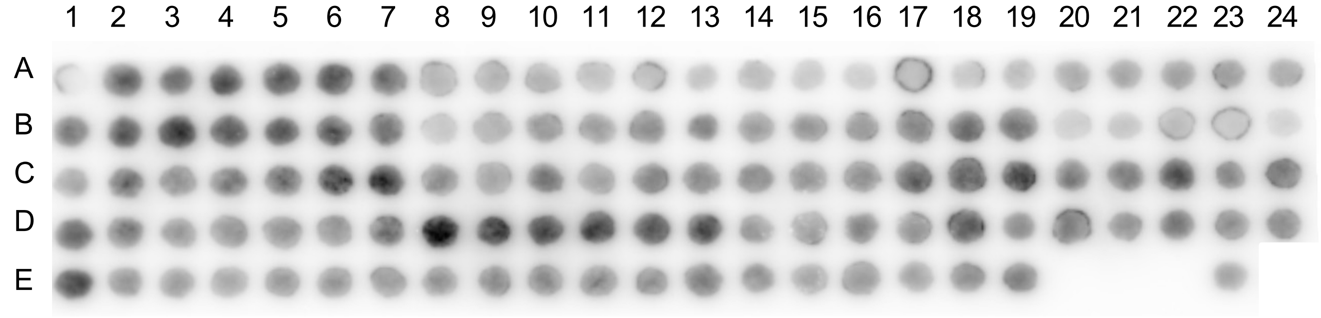

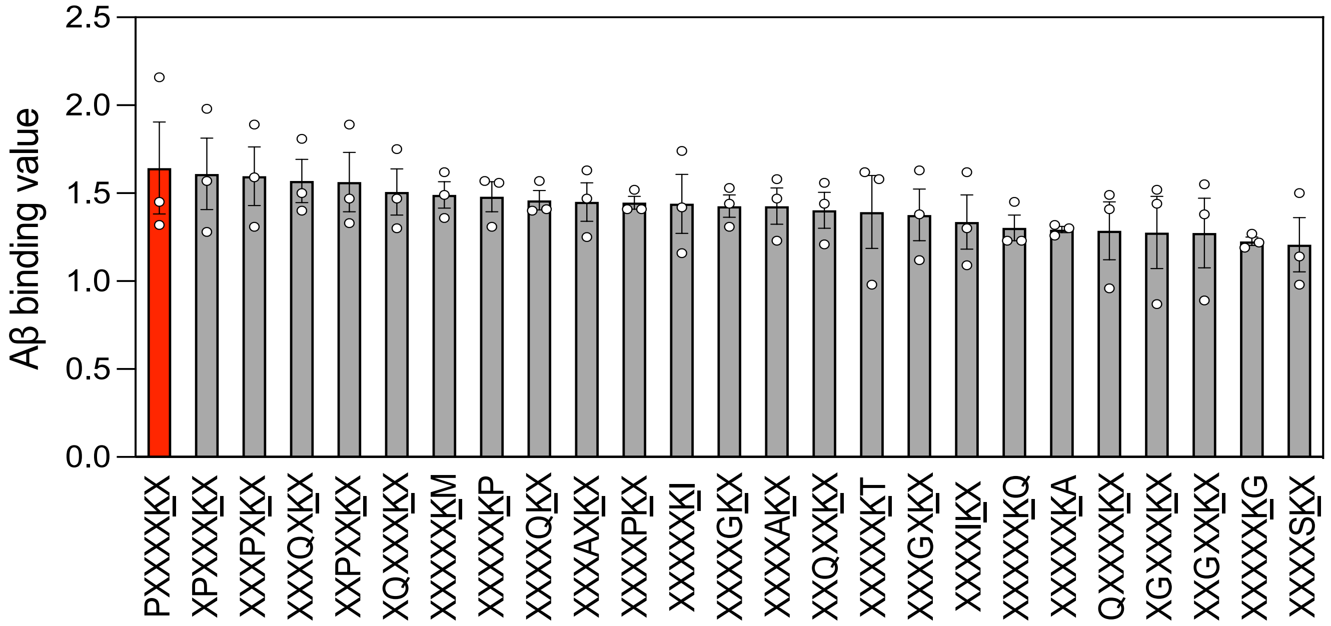

Supplementary Fig. 1c

3rd screening

2 3 4 5 7

PXXXXKX-U

	1	2	3	4	5	6	7	8	9	10	11	12	13	14	15	16	17	18	19	20	21	22	23	24
A	2A	3A	4A	5A	7A	2R	3R	4R	5R	7R	2N	3N	4N	5N	7N	2D	3D	4D	5D	7D	2Q	3Q	4Q	5Q
B	7Q	2E	3E	4E	5E	7E	2G	3G	4G	5G	7G	2H	3H	4H	5H	7H	2I	3I	4I	5I	7I	2L	3L	4L
C	5L	7L	2K	3K	4K	5K	7K	2M	3M	4M	5M	7M	2F	3F	4F	5F	7F	2P	3P	4P	5P	7P	2S	3S
D	4S	5S	7S	2T	3T	4T	5T	7T	2W	3W	4W	5W	7W	2Y	3Y	4Y	5Y	7Y	2V	3V	4V	5V	7V	ori

ori: original motif; PXXXXKX-U

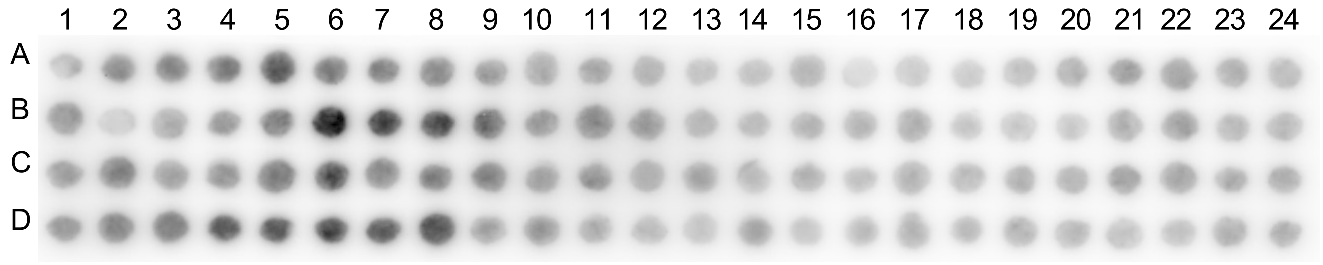

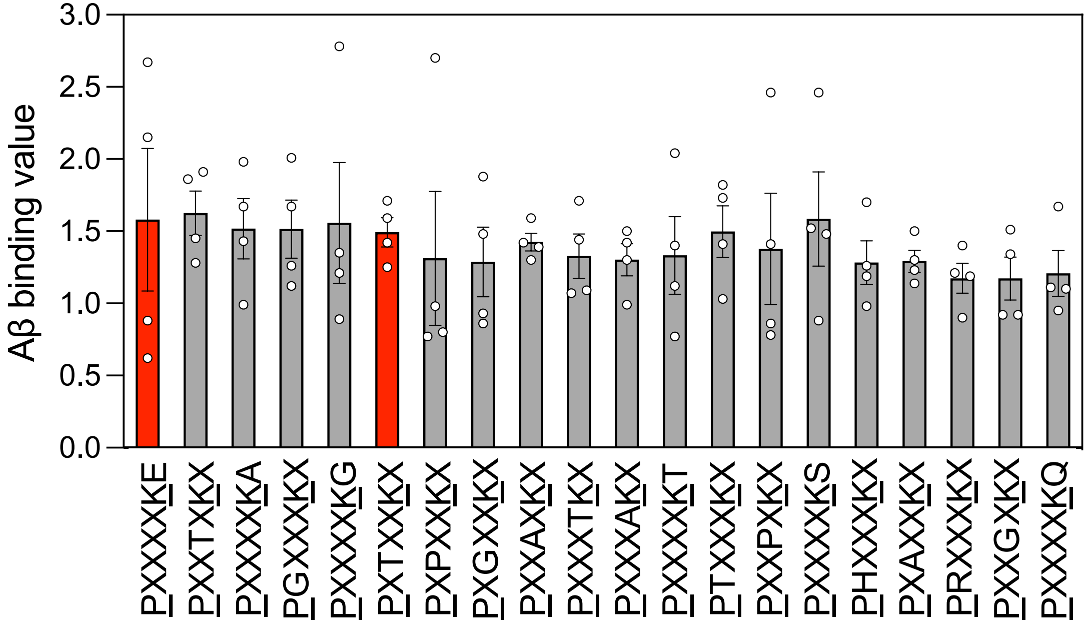

Supplementary Fig. 1d

4th screening (Group I)

2 3 4 5

PXXXXKE-U

	1	2	3	4	5	6	7	8	9	10	11	12	13	14	15	16	17	18	19	20	21	22	23	24
A																2A	3A	4A	5A	2R	3R	4R	5R	2N
B	3N	4N	5N	2D	3D	4D	5D	2Q	3Q	4Q	5Q	2E	3E	4E	5E	2G	3G	4G	5G	2H	3H	4H	5H	2I
C	3I	4I	5I	2L	3L	4L	5L	2K	3K	4K	5K	2M	3M	4M	5M	2F	3F	4F	5F	2P	3P	4P	5P	2S
D	3S	4S	5S	2T	3T	4T	5T	2W	3W	4W	5W	2Y	3Y	4Y	5Y	2V	3V	4V	5V	ori				

ori: original motif; PXXXXKE-U

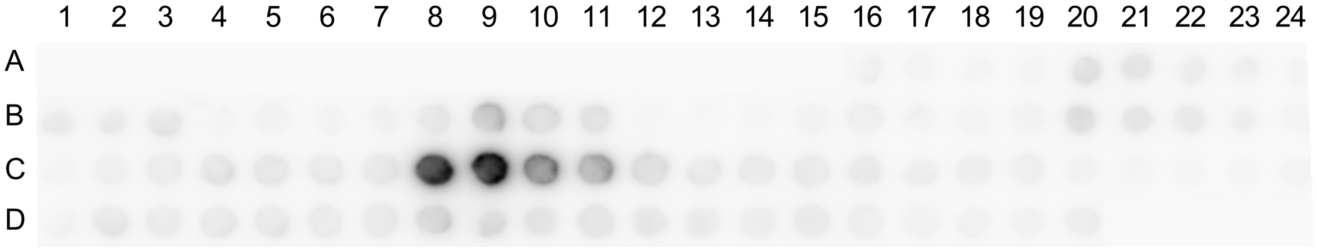

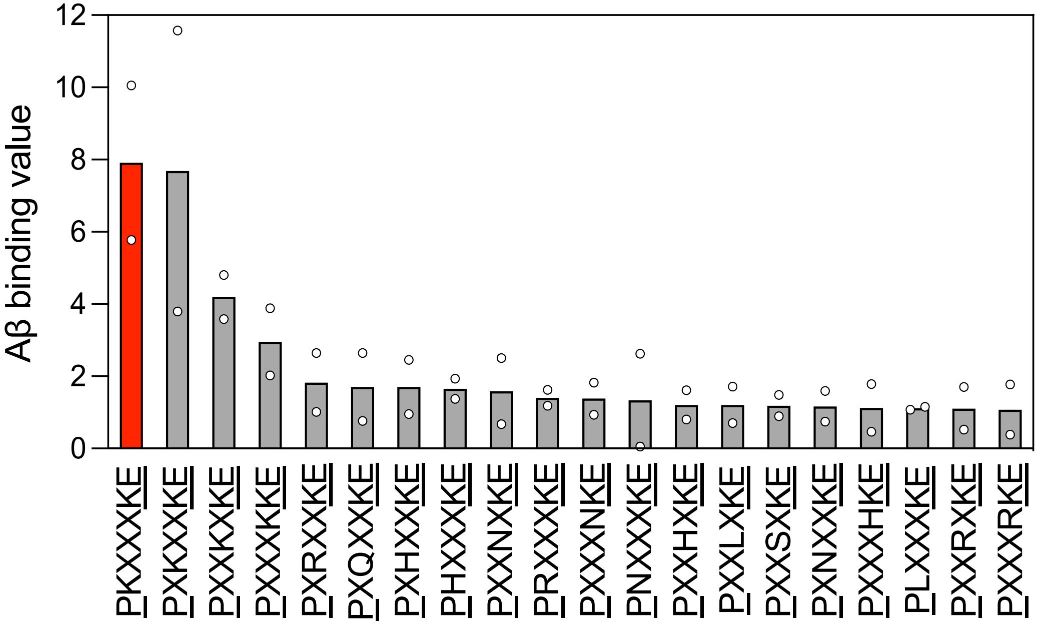

Supplementary Fig. 1e

4th screening (Group II)

2 4 5 7

PXTXXKX-U

	1	2	3	4	5	6	7	8	9	10	11	12	13	14	15	16	17	18	19	20	21	22	23	24
A											2A	4A	5A	7A	2R	4R	5R	7R	2N	4N	5N	7N	2D	4D
B	5D	7D	2Q	4Q	5Q	7Q	2E	4E	5E	7E	2G	4G	5G	7G	2H	4H	5H	7H	2I	4I	5I	7I	2L	4L
C	5L	7L	2K	4K	5K	7K	2M	4M	5M	7M	2F	4F	5F	7F	2P	4P	5P	7P	2S	4S	5S	7S	2T	4T
D	5T	7T	2W	4W	5W	7W	2Y	4Y	5Y	7Y	2V	4V	5V	7V	ori									

ori: original motif; PXTXXKX-U

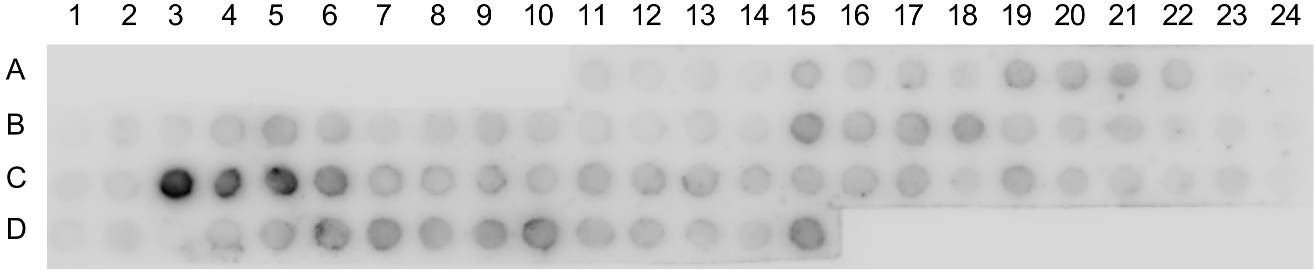

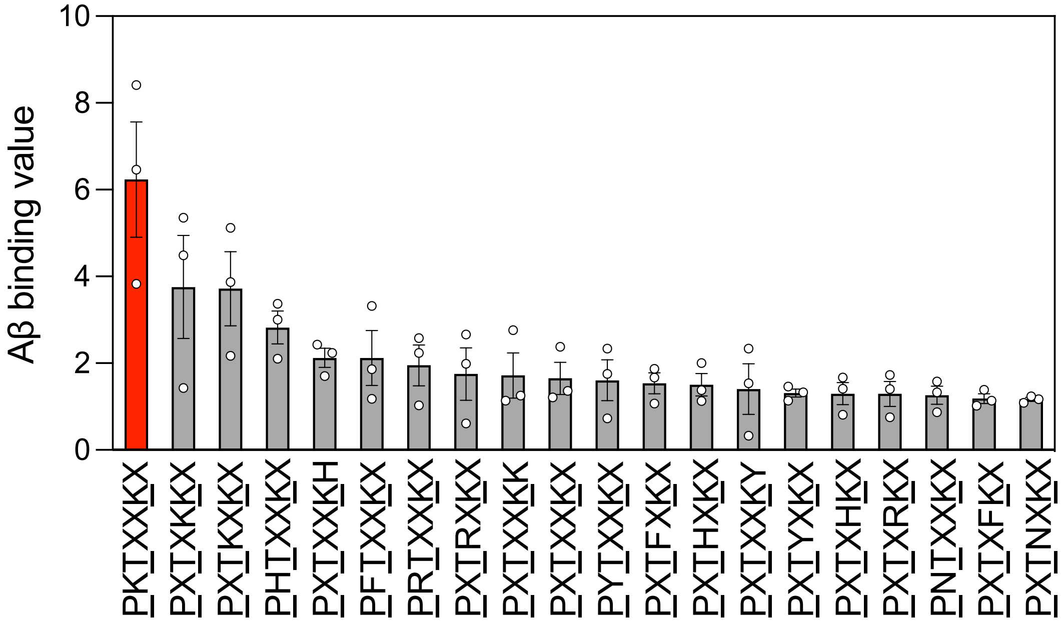

Supplementary Fig. 1f

5th screening (Group I)

3 4 5

PKXXXKE-U

	1	2	3	4	5	6	7	8	9	10	11	12	13	14	15	16	17	18	19	20	21	22	23	24
A																	3A	4A	5A	3R	4R	5R	3N	4N
B	5N	3D	4D	5D	3Q	4Q	5Q	3E	4E	5E	3G	4G	5G	3H	4H	5H	3I	4I	5I	3L	4L	5L	3K	4K
C	5K	3M	4M	5M	3F	4F	5F	3P	4P	5P	3S	4S	5S	3T	4T	5T	3W	4W	5W	3Y	4Y	5Y	3V	4V
D	5V	ori																						

ori: original motif; PKXXXKE-U

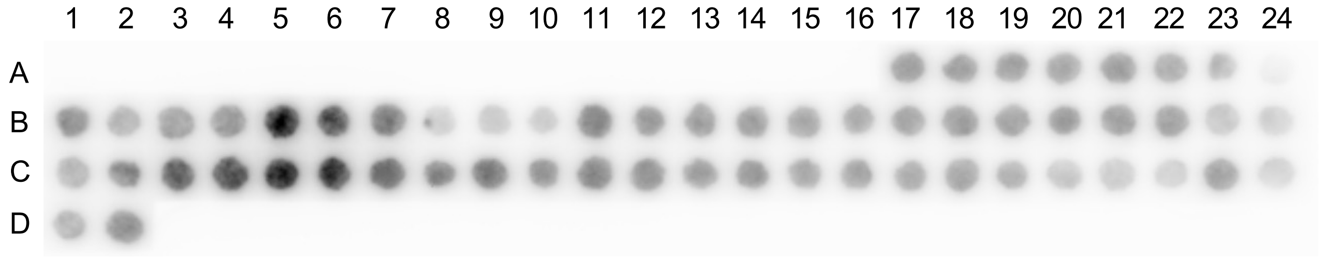

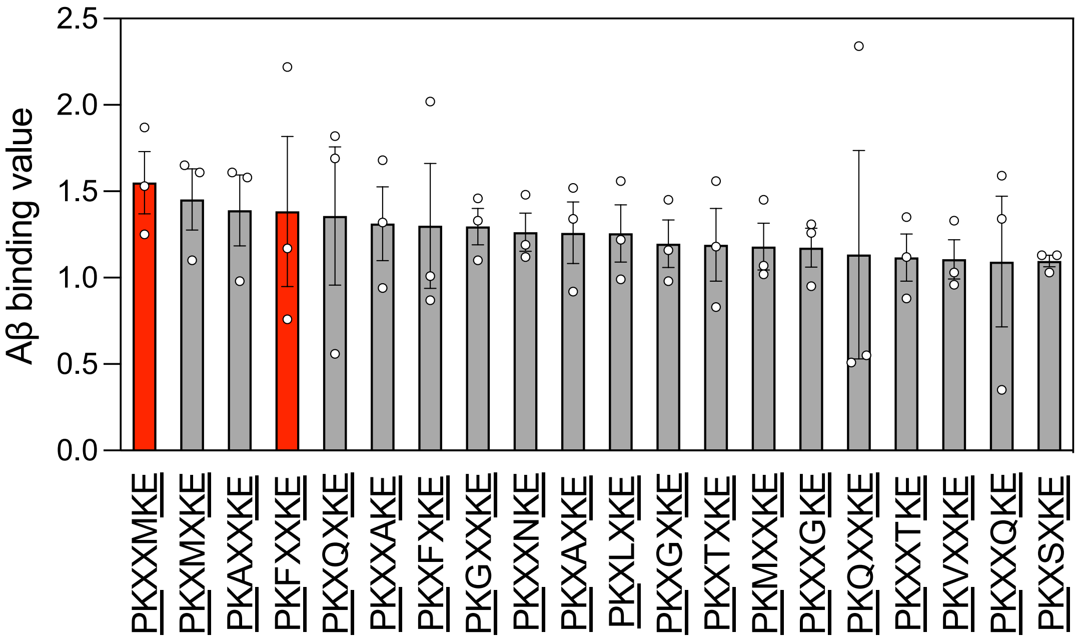

Supplementary Fig. 1g

5th screening (Group II)

4 5 7

PKTXXKX-U

	1	2	3	4	5	6	7	8	9	10	11	12	13	14	15	16	17	18	19	20	21	22	23	24
A																					4A	5A	7A	4R
B	5R	7R	4N	5N	7N	4D	5D	7D	4Q	5Q	7Q	4E	5E	7E	4G	5G	7G	4H	5H	7H	4I	5I	7I	4L
C	5L	7L	4K	5K	7K	4M	5M	7M	4F	5F	7F	4P	5P	7P	4S	5S	7S	4T	5T	7T	4W	5W	7W	4Y
D	5Y	7Y	4V	5V	7V	ori																		

ori: original motif; PKTXXKX-U

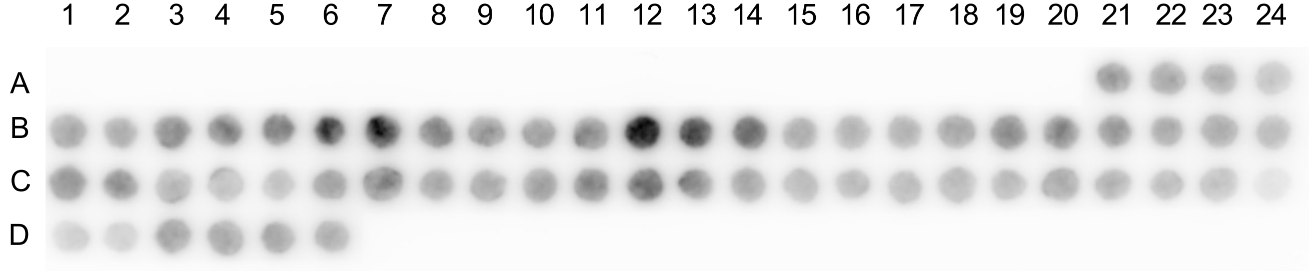

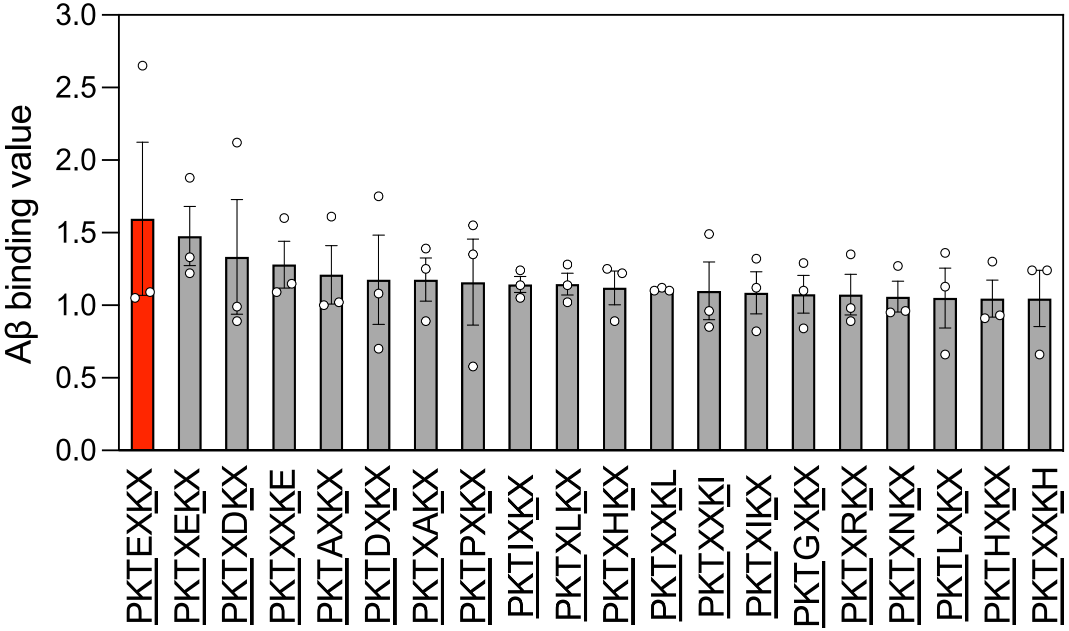

6th screening (Group Ia)

3 4

PKXXMKE-U

Supplementary Fig. 1h

	1	2	3	4	5	6	7	8
A								3A
B	4A	3R	4R	3N	4N	3D	4D	3Q
C	4Q	3E	4E	3G	4G	3H	4H	3I
D	4I	3L	4L	3K	4K	3M	4M	3F
E	4F	3P	4P	3S	4S	3T	4T	3W
F	4W	3Y	4Y	3V	4V	ori		

ori: original motif; PKXXMKE-U

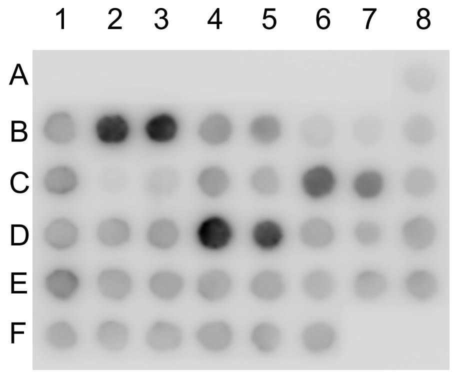

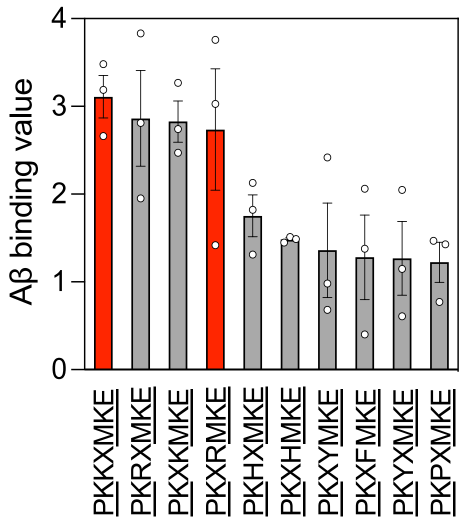

Supplementary Fig. 1i

6th screening (Group Ib)

45

PKFXXKE-U

	1	2	3	4	5	6	7	8
A	4A	5A	4R	5R	4N	5N	4D	5D
B	4Q	5Q	4E	5E	4G	5G	4H	5H
C	4I	5I	4L	5L	4K	5K	4M	5M
D	4F	5F	4P	5P	4S	5S	4T	5T
E	4W	5W	4Y	5Y	4V	5V	ori	

ori: original motif; PKFXXKE-U

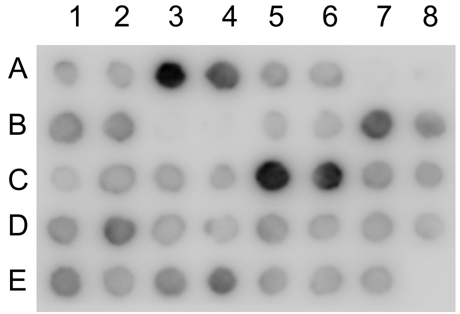

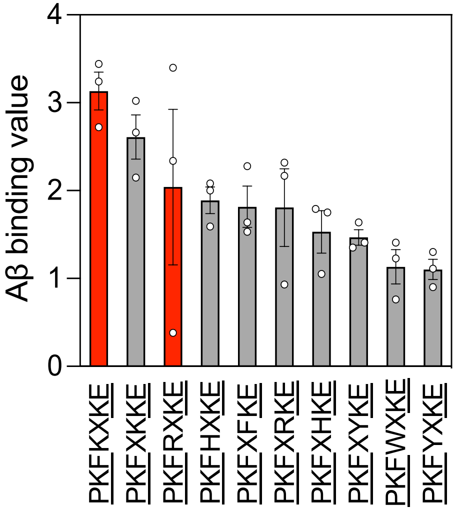

Supplementary Fig. 1j

6th screening （Group II）

5 7

PKTEXKX-U

	1	2	3	4	5	6	7	8
A							5A	7A
B	5R	7R	5N	7N	5D	7D	5Q	7Q
C	5E	7E	5G	7G	5H	7H	5I	7I
D	5L	7L	5K	7K	5M	7M	5F	7F
E	5P	7P	5S	7S	5T	7T	5W	7W
F	5Y	7Y	5V					
G	7V	ori						

ori: original motif; PKTEXKX-U

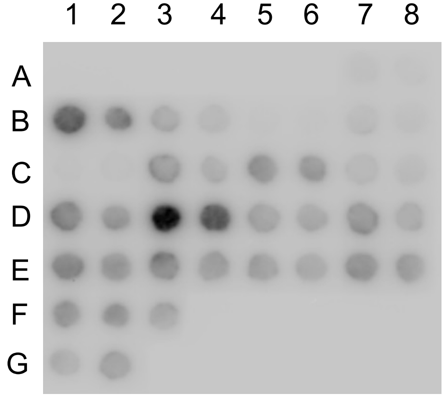

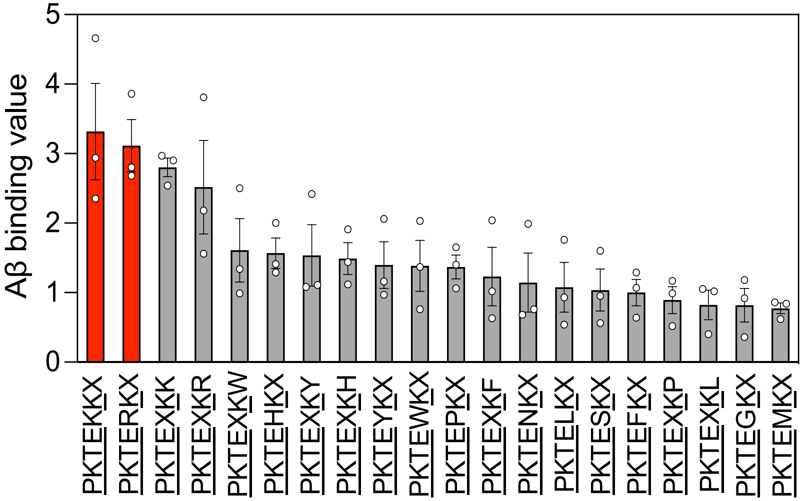

Supplementary Fig. 1: Affinity-driven screening of tetravalent peptide libraries synthesized on a membrane.

a The library for the first round of screening contained tetravalent peptides with the following sequence X-X-X-X-X-X-X-U- (where X is a mixture of all amino acids except Cys, and U is amino hexanoic acid as a spacer), and each X was replaced individually by a fixed amino acid, excluding Cys. Each cell indicates the position and the amino acid replaced with an X. Membranes were blotted with biotinylated Aβ1-28 (10 μg/ml; upper panel). The bound Aβ1-28 to each tetravalent peptide was quantified as a pixel value as described in Methods. The sum of the pixel value of all peptide spots was normalized to 133 (the number of tetravalent peptides synthesized on the membrane) so that each peptide would have a value 1 in the absence of selectivity for Aβ1-28. Each screen was performed twice; a representative blotted membrane is shown. The motifs were sorted in descending order based on the binding values. The binding values of the top 30 motifs are shown (lower panel). Based on the results, the second library (**b**) was designed as follows.

b The library for the second round of screening contained tetravalent peptides with the following sequence, X-X-X-X-X-<u>K</u>-X-U-. The membrane was analyzed as described above (mean ± SE, n = 3). Each screen was performed three times; a representative blotted membrane is shown. The binding values of the top 25 motifs are shown (lower panel). Based on the results, the third library (**c**) was designed as follows.

c The library for the third round of screening contained tetravalent peptides with the following sequence, <u>P</u>-X-X-X-X-<u>K</u>-X-U-. The membrane was analyzed as described above (mean ± SE, n = 3). Each screen was performed three times; a representative blotted membrane is shown. The binding values of the top 20 motifs are shown (lower panel). Based on the results, the fourth libraries (**d** and **e**) were designed as follows.

d The library for the fourth round of screening contained tetravalent peptides with the following sequence, P-X-X-X-X-K-E-U- (4th library, Group I). Each membrane was analyzed as described above (mean ± SE, *n* = 2). Each screen was performed three times; a representative blotted membrane is shown. The binding values of the top 20 motifs are shown (lower panel). Based on the results, the fifth library (**f**) was designed as follows.
e The library for the fourth round of screening contained tetravalent peptides with the following sequence, P-X-T-X-X-K-X-U- (4th library, Group II). Each membrane was analyzed as described above (mean ± SE, *n* = 3). Each screen was performed three times; a representative blotted membrane is shown. The binding values of the top 20 motifs are shown (lower panel). Based on the results, the fifth library (**g**) was designed as follows.
f The library for the fifth round of screening contained tetravalent peptides with the following sequence, P-K-X-X-X-K-E-U- (5th library, Group I). Each membrane was analyzed as described above (mean ± SE, *n* = 3). Each screen was performed three times; a representative blotted membrane is shown. The binding values of the top 20 motifs are shown (lower panel). Based on the results, the sixth libraries (**h** and **i**) were designed as follows.
g The library for the fifth round of screening contained tetravalent peptides with the following sequence, P-K-T-X-X-K-X-U- (5th library, Group II). Each membrane was analyzed as described above (mean ± SE, *n* = 3). Each screen was performed three times; a representative blotted membrane is shown. The binding values of the top 20 motifs are shown (lower panel). Based on the results, the sixth library (**j**) was designed as follows.

h The library for the sixth round of screening contained tetravalent peptides with the following sequence, P-K-X-X-M-K-E-U- (6th library, Group Ia). Each membrane was analyzed as described above (mean ± SE, *n* = 3). Each screen was performed three times; a representative blotted membrane is shown. The binding values of the top 10 motifs are shown (lower panel). Based on the results, the seventh libraries were designed (Fig. 1c).
i The library for the sixth round of screening contained tetravalent peptides with the following sequence, P-K-F-X-X-K-E-U- (6th library, Group Ib). Each membrane was analyzed as described above (mean ± SE, *n* = 3). Each screen was performed three times; a representative blotted membrane is shown. The binding values of the top 10 motifs are shown (lower panel). Based on the results, the seventh libraries were designed (Fig. 1c).
j The library for the sixth round of screening contained tetravalent peptides with the following sequence, P-K-T-E-X-K-X-U- (6th library, Group II). Each membrane was analyzed as described above (mean ± SE, *n* = 3). Each screen was performed three times; a representative blotted membrane is shown. The binding values of the top 10 motifs are shown (lower panel). Based on the results, the seventh libraries were designed (Fig. 1c).

Supplementary Fig. 2

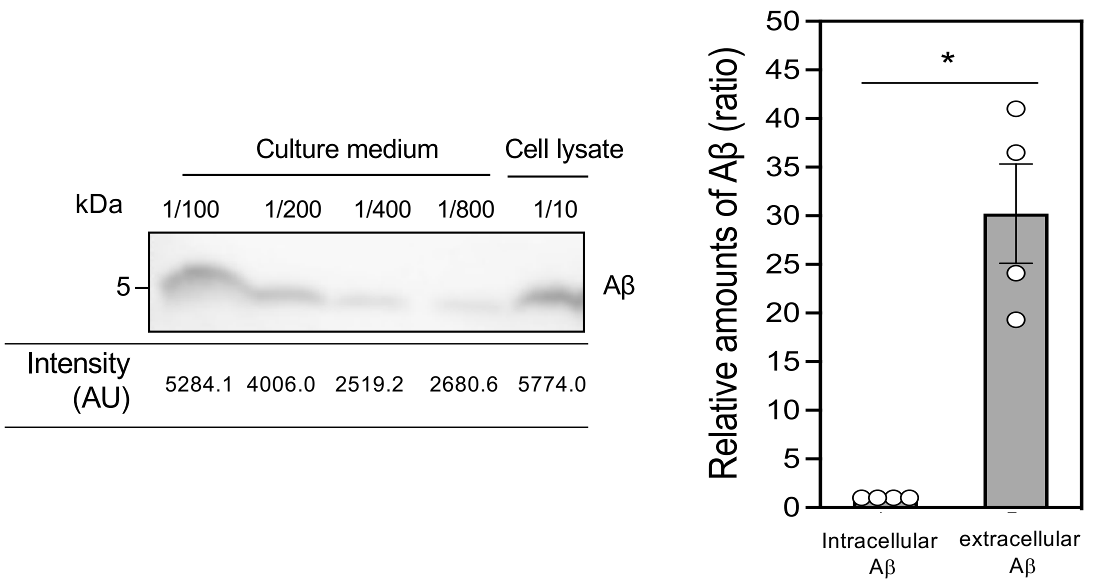

Supplementary Fig. 2: Comparison between the amount of extracellular Aβ and that of intracellular Aβ.

7WD10 cells were cultured for 48 h; the culture medium (for extracellular Aβ) and cell lysates (for intracellular Aβ) were analyzed by western blotting. The volumes of the culture medium or cell lysates used are indicated. The total amounts of Aβ were measured using Image J software (NIH). We performed four independent experiments, and representative data are shown (left panel). Relative amounts of Aβ (right panel). Data are presented as a ratio to the amount of intracellular Aβ (mean ± SE, n = 4). Significance was calculated by two-sided Student's t test, *P <0.05.

Supplementary Fig. 3

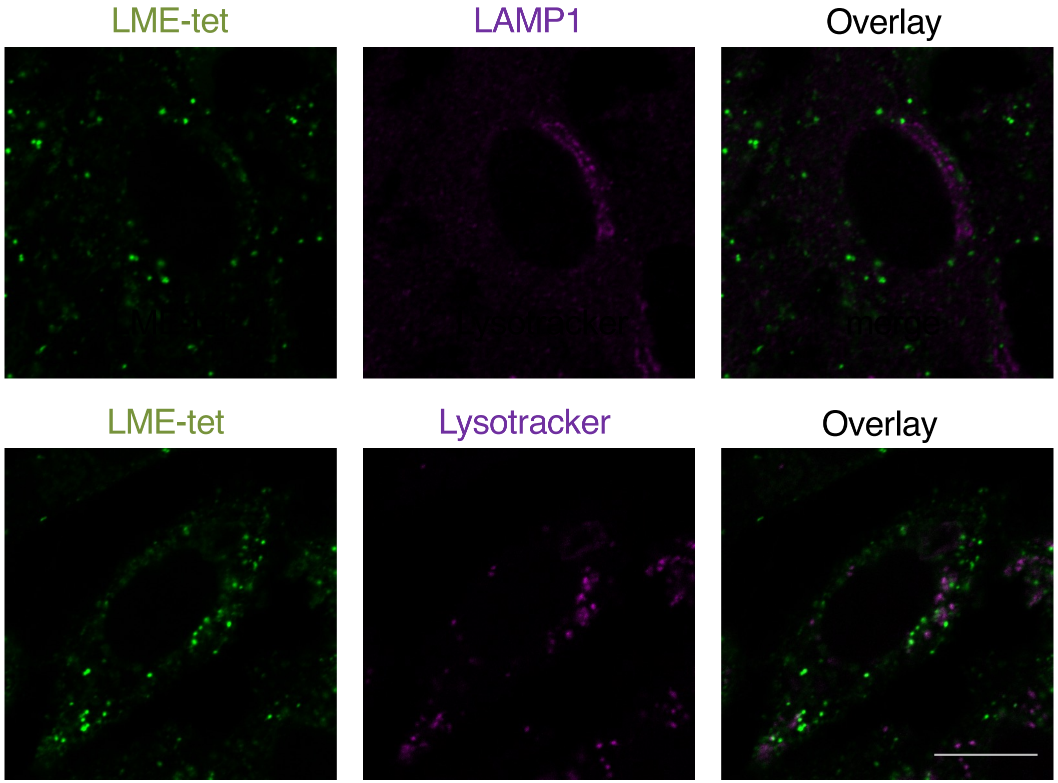

Supplementary Fig. 3: LME-tet co-localization with lysosome markers.

LME-tet co-localization with lysosome-associated membrane glycoprotein 1 (LAMP1), a lysosomal marker, was analyzed by immunocytochemical staining with rabbit polyclonal anti-LAMP1 antibody, followed by Alexa Fluor 488-conjugated goat anti-rabbit IgG antibody (upper panel). Acidification of LME-tet-containing endosomes was analyzed using Lysotracker (lower panel). In both cases, 7WD10 cells were treated with biotinylated LME-tet (50 μM) for 60 min at 37°C, and LME-tet was detected by Alexa Fluor 488-conjugated streptavidin. The scale bar indicates 10 μm.

Supplementary Fig. 4a

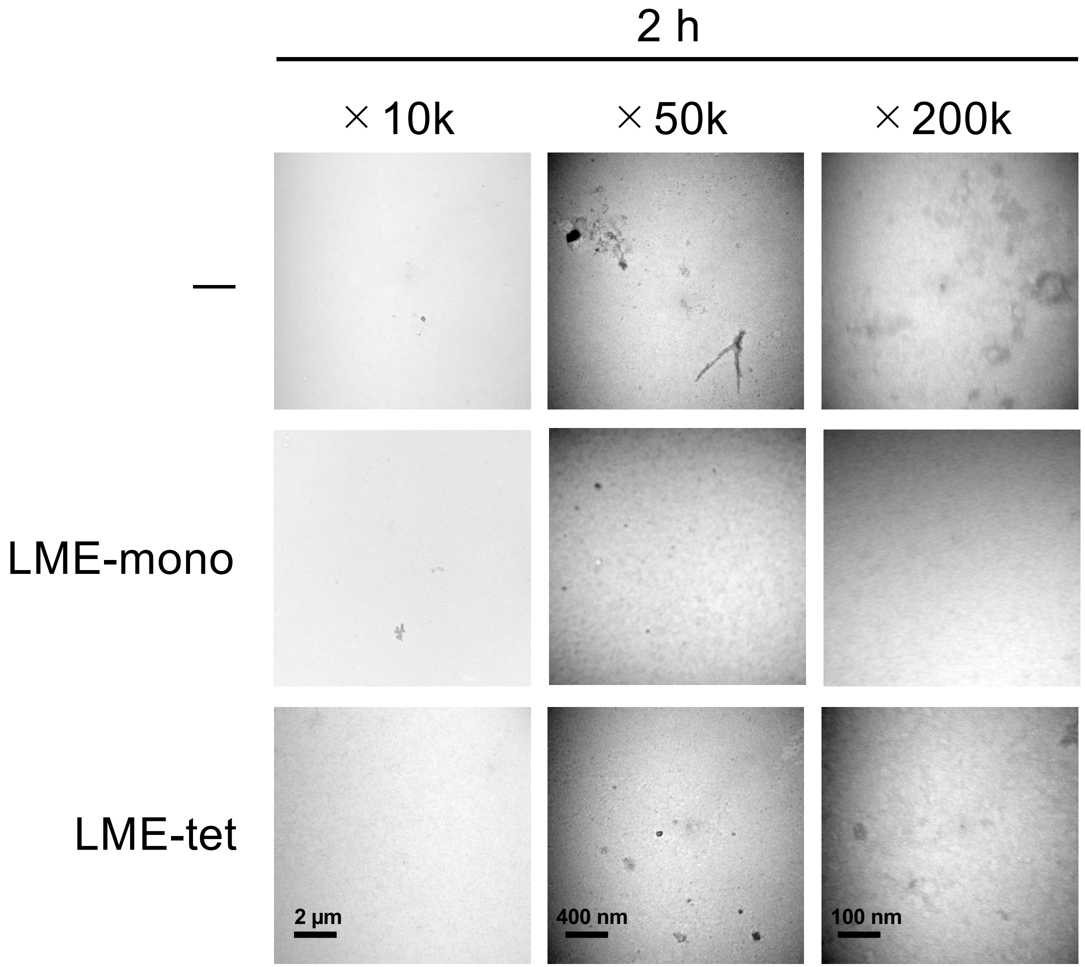

Supplementary Fig. 4b

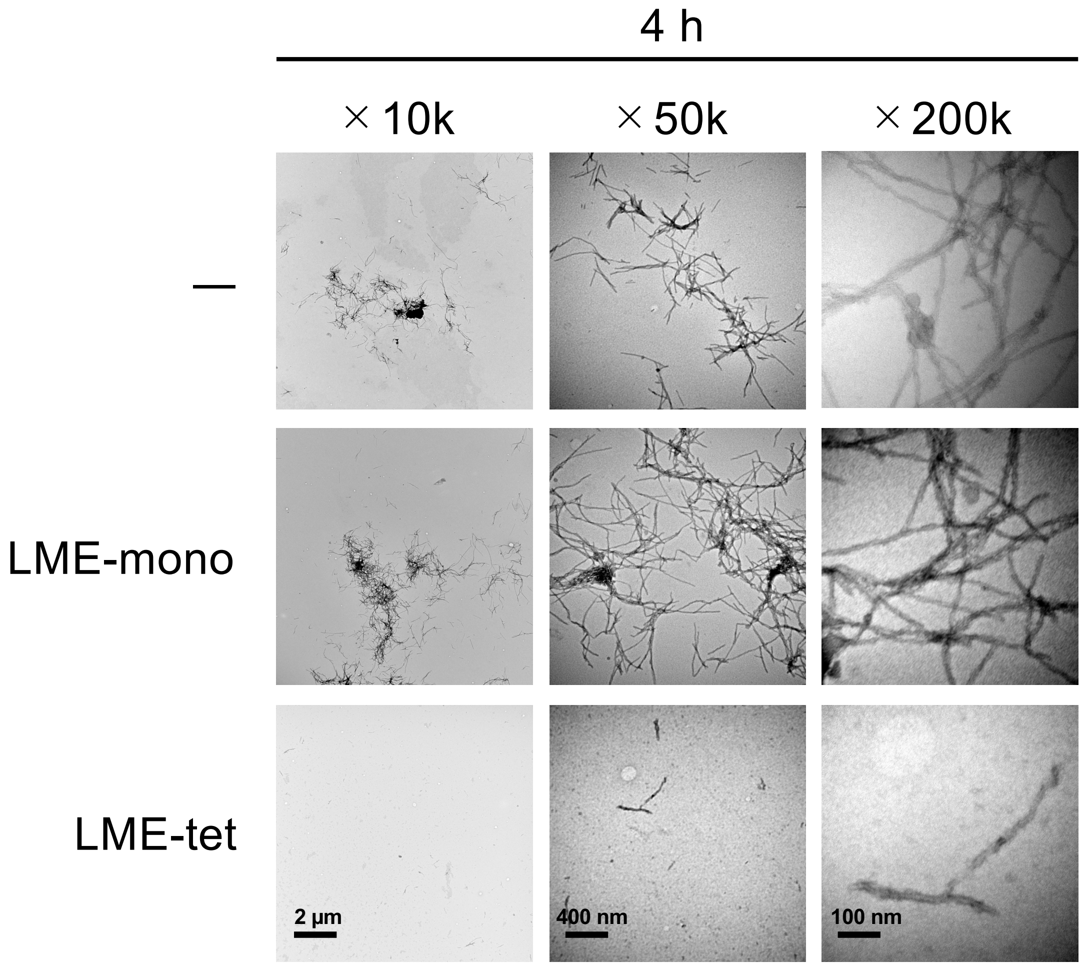

Supplementary Fig. 4c

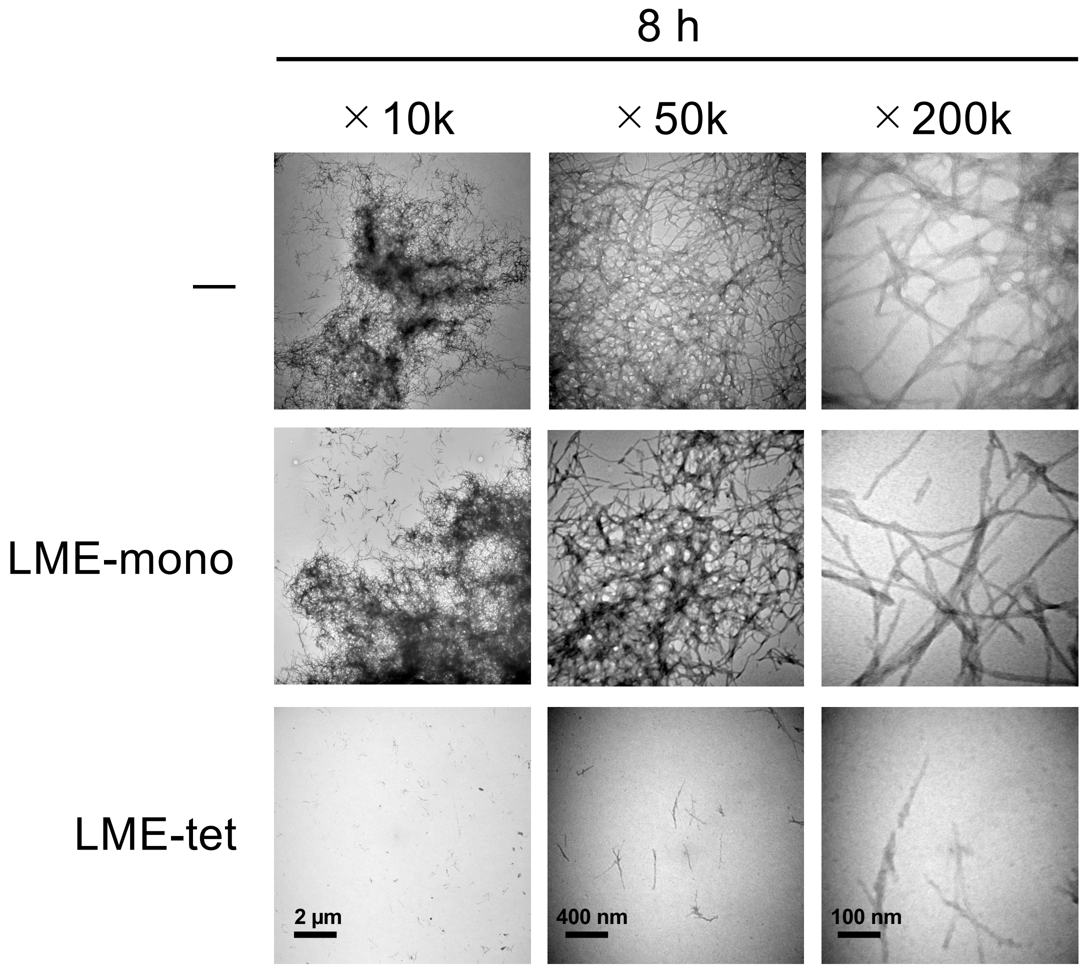

Supplementary Fig. 4d

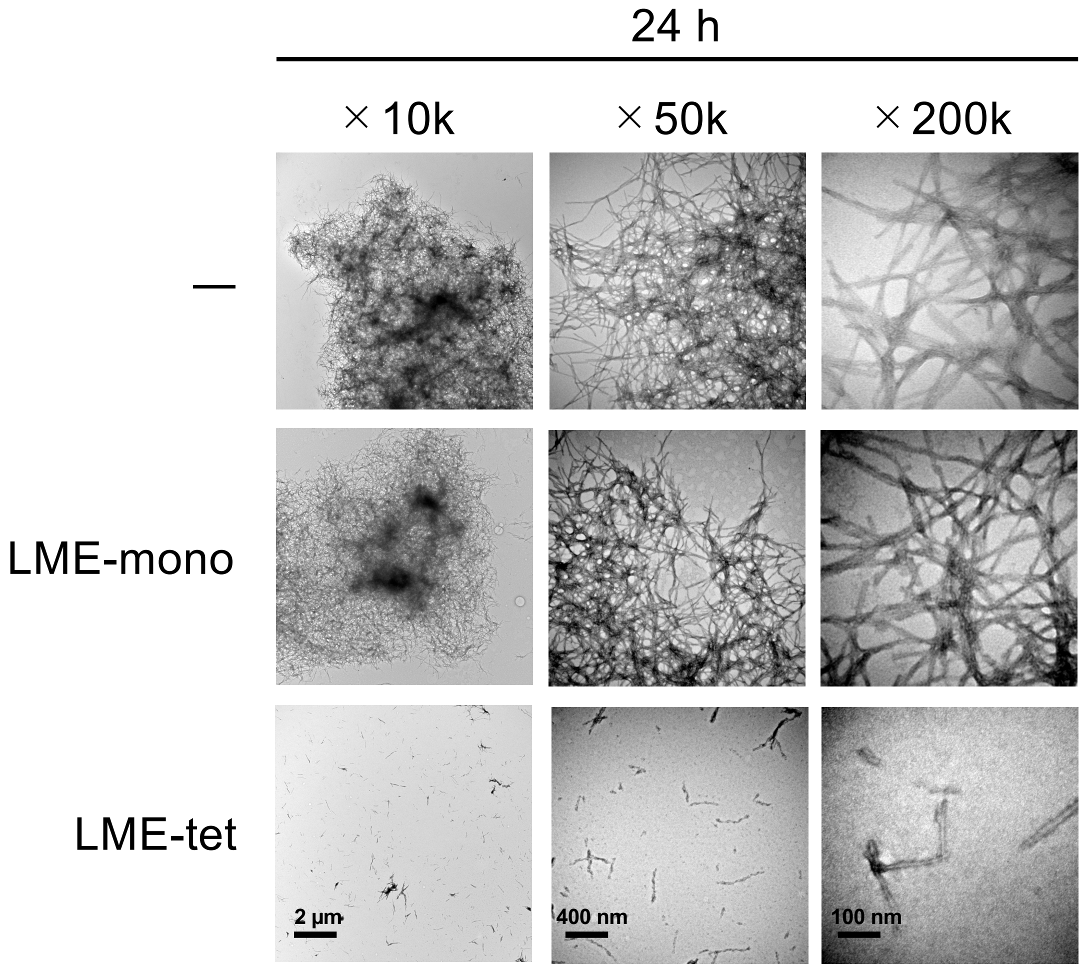

Supplementary Fig. 4: Time-dependent fibrillation profiles of Aβ42.

Aβ42 (20 μM) was incubated in the presence or absence of each peptide at 37 ºC for the indicated times. Fibrillation profiles of Aβ42 were analyzed by electron microscopy. Representative images at ×10K, ×50K, and ×200K magnification (left, center, and right, respectively) are shown. Scale bars indicate 2 μm, 400 nm, and 100 nm, respectively.

Supplementary Fig. 5

a

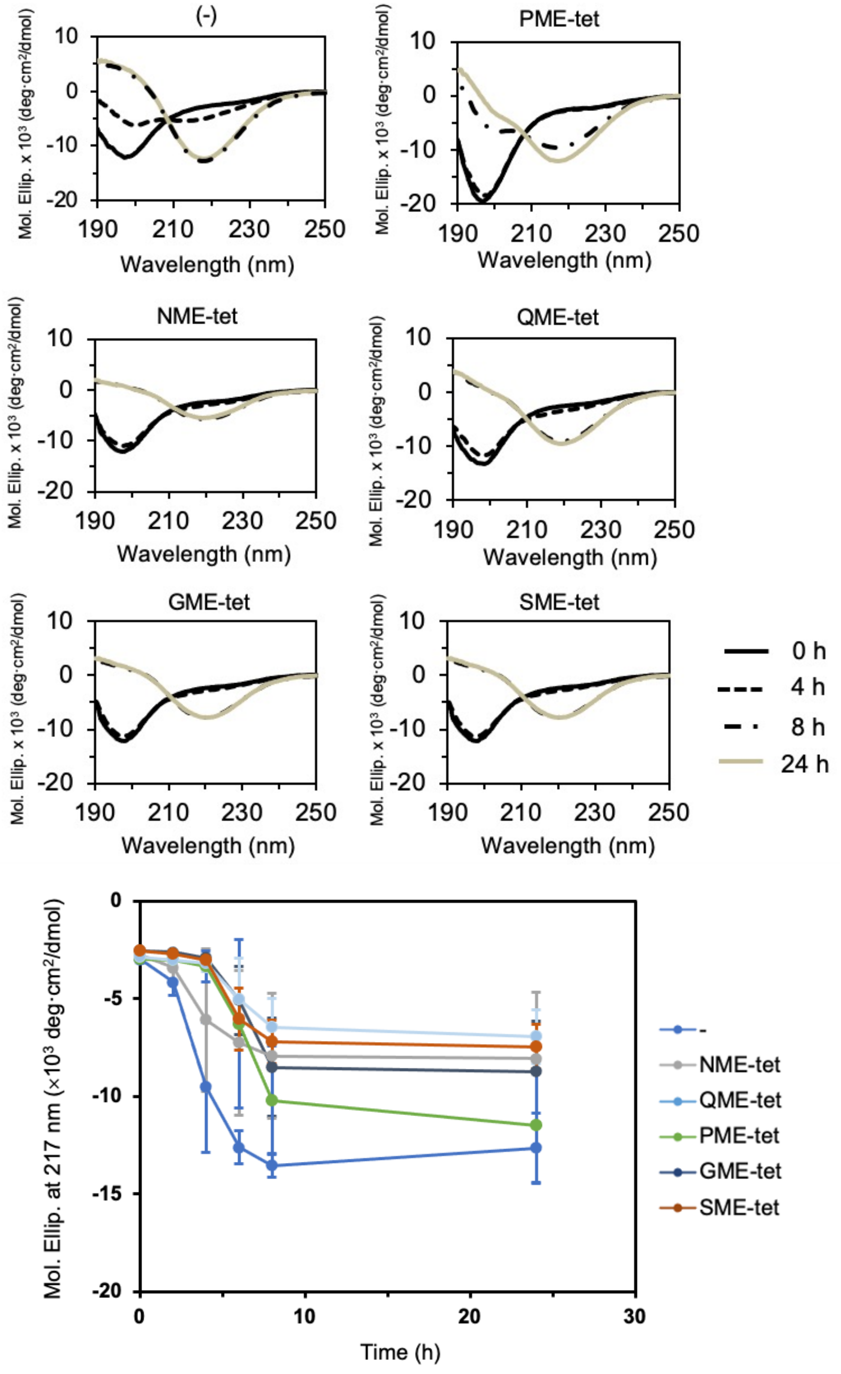

b

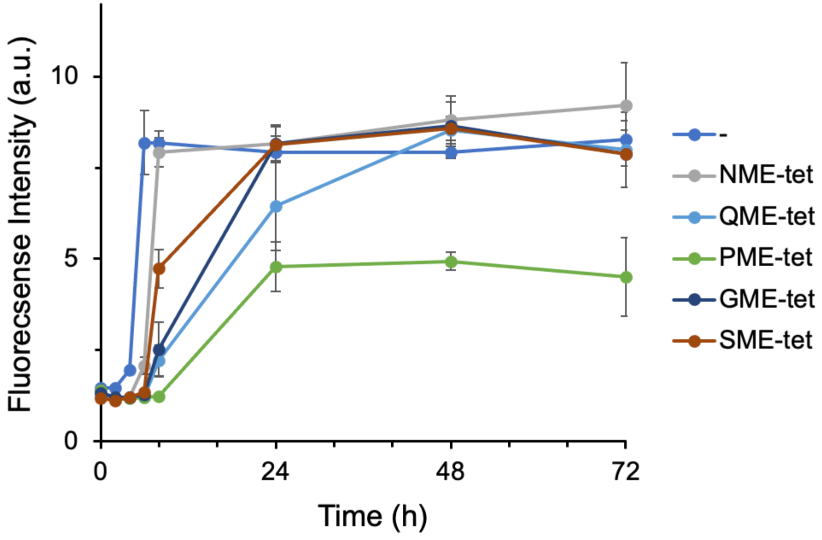

Supplementary Fig. 5: Effects of various tetravalent peptides on the conformational change and subsequent fibrillation of Aβ42.

a Time-dependent conformational change in Aβ42 (20 μM) in the presence or absence of various tetravalent peptides (2 μM). Far-UV CD spectra (wavelength 190-250 nm) were measured at the indicated time periods. Kinetic rates of β-sheet formation of Aβ42 were estimated by measuring time-dependent alterations of molar ellipticities of CD spectra at 217 nm. Data represent mean ± SE, $n = 3$-4. **b** Fibrillation profiles of Aβ42 in the presence or absence of various tetravalent peptides (2 μM), measured by the Thioflavin T-binding assay. Time-dependent increments of Aβ42-Thioflavin T binding are shown. Data represent mean ± SE, $n = 3$.

Supplementary Fig. 6

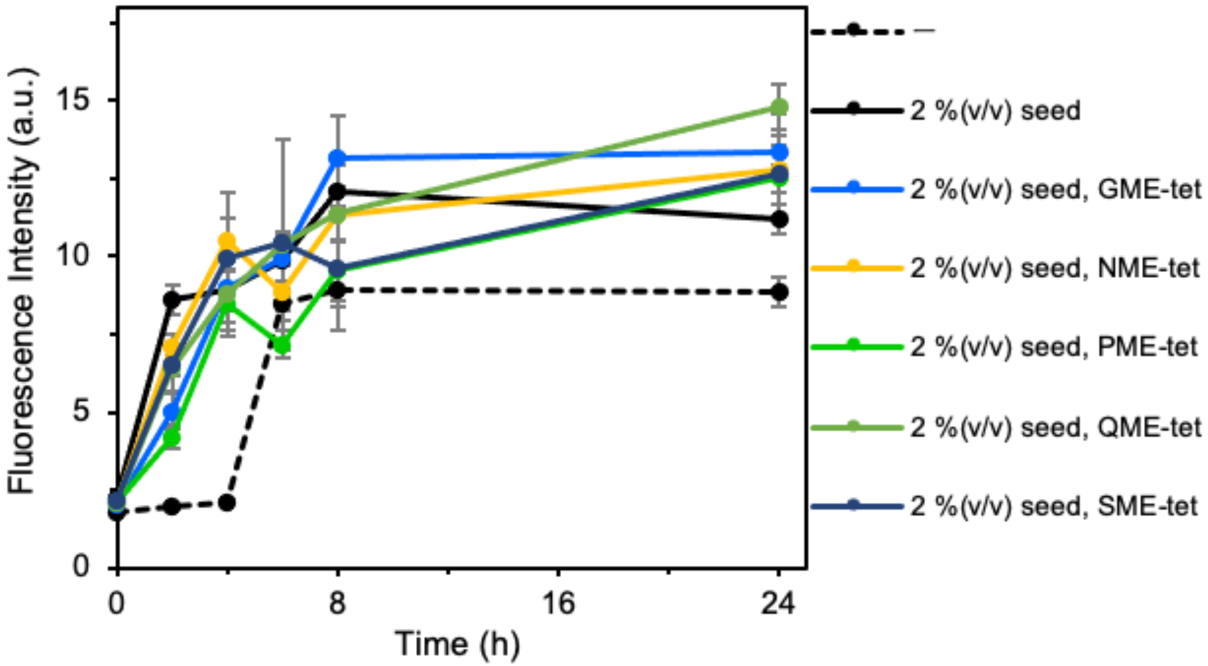

Supplementary Fig. 6: Effects of various tetravalent peptides on growth of Aβ42 fibril.
Effects of various tetravalent peptides (2 μM) on growth of Aβ42 fibril in the presence of 2 % (v/v) Aβ42 fibril-seed, measured by the Thioflavin T-binding assay. Time-dependent increments of Aβ42-Thioflavin T binding are shown. Data represent mean ± SE, $n = 3$.

Supplementary Fig. 7

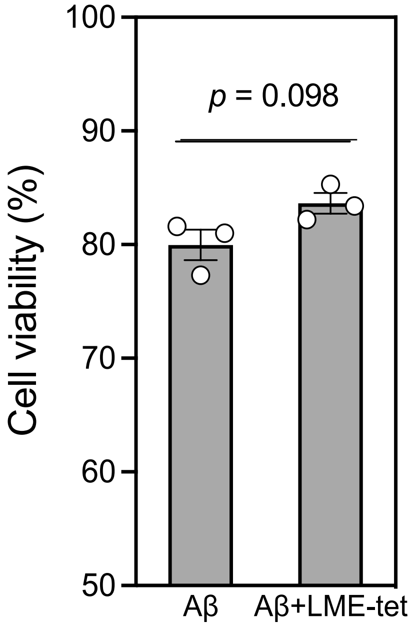

Supplementary Fig. 7: The effect of Ac-LME-tet on the cytotoxicity of Aβ42 in Neuro-2a cells.

Neuro-2a cells （CCL-131, ATCC） were maintained at 37°C in Dulbecco's Modified Eagle Medium (DMEM; Sigma) supplemented with 10% fetal bovine serum (FBS), 50 units/ml penicillin, and 50 μg/ml streptomycin. Neuro-2a cells were treated with Aβ42 (10 μM) in the presence or absence of Ac-LME-tet (80 μM) at 37°C for 48 h. The relative numbers of living cells were determined using a cell counting reagent (Nacalai Tesque, Japan). Data are presented as a percentage of the control value without Ac-LME-tet (mean ± SE, n = 3). Significant differences were analyzed using an unpaired two-sided Student's t-test.

Supplementary Fig. 8

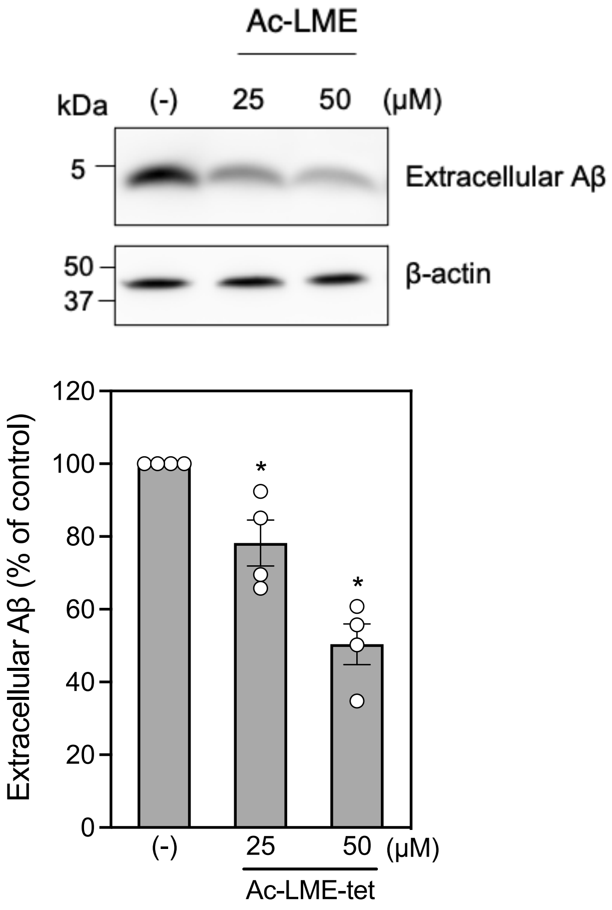

Supplementary Fig. 8: The effect of Ac-LME-tet on Aβ production. 7WD10 cells were treated with vehicle control or Ac-LME-tet at the indicated concentrations for 48 h, and the culture medium was analyzed by western blot. Data are graphed as the percentage of the control value, showing the mean ± SE; $n = 3$. Significance *vs*. vehicle was calculated by ANOVA, followed by one-sided Dunnett's test; $^*P < 0.05$.

Supplementary Fig. 9

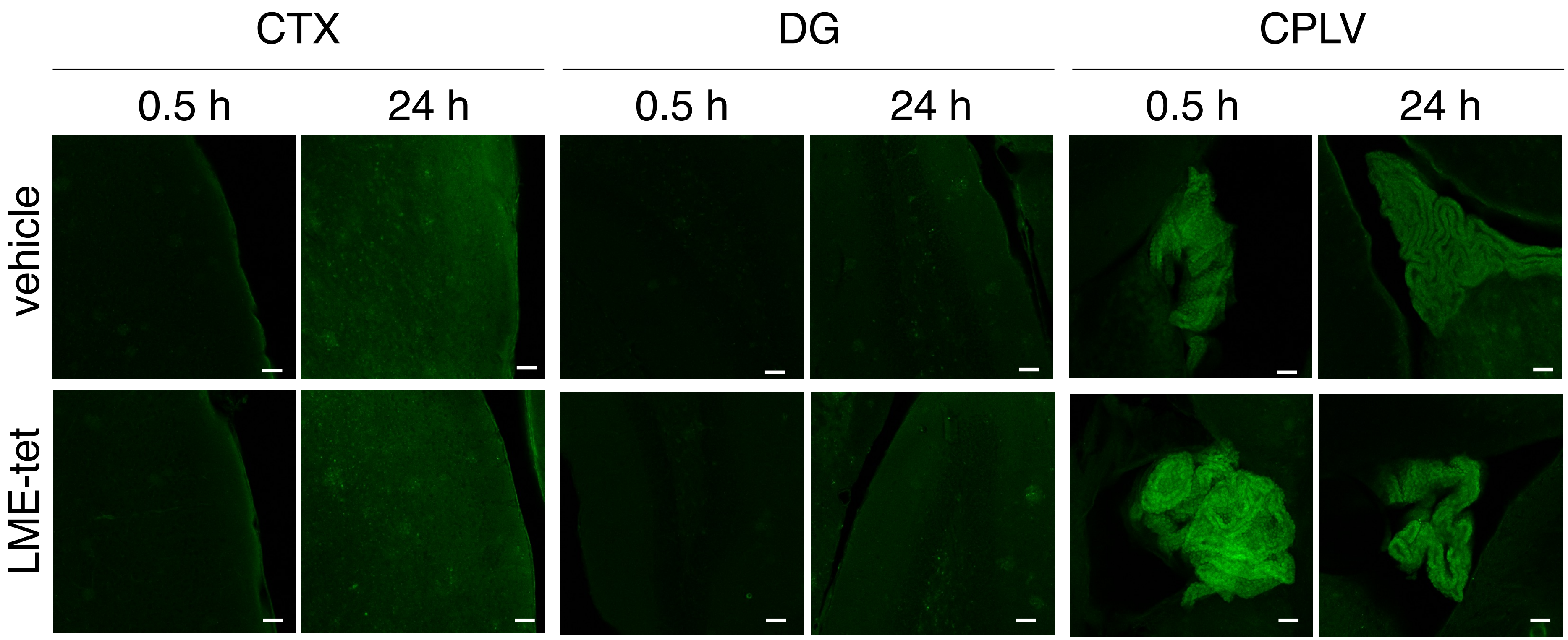

Supplementary Fig. 9: Detection of LME-tet in the brain.

Fluorescein isothiocyanate (FITC)-labelled LME-tet was administered to *App*$^{NL\text{-}G\text{-}F/NL\text{-}G\text{-}F}$ mice intraperitoneally (71 mg/kg). After the indicated times, mice were sacrificed, and the brains were removed. After perfusion fixation, brains were fixed in 10% formaldehyde for 2 days, and 100-μm-thick coronal sections processed by a brain slicer (Linearslicer Pro7) were examined via confocal scanning laser microscopy to visualize the peptide. The cerebral cortex (CTX), dentate gyrus (DG), and choroid plexus of the lateral ventricle (CPLV) from vehicle control mice and peptide-treated mice were analyzed. Scale bars indicate 50 μm.

Supplementary Fig. 10

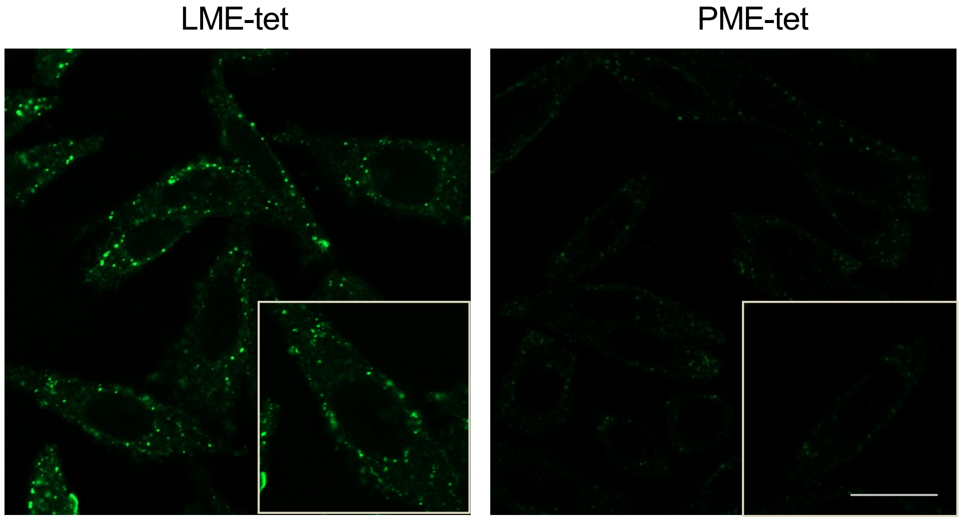

Supplementary Fig. 10: Efficient incorporation of PME-tret into cells.
7WD10 cells were cultured at 37°C for 1 h in the presence of biotinylated LME-tet or PME-tet (50 μM). Biotinylated peptide was detected using Alexa Fluor 488-conjugated streptavidin. Fluorescence images were obtained using confocal laser scanning microscopy to assess the incorporation of tetravalent peptides into cells. The scale bars indicate 10 μm.

Supplementary Fig. 11

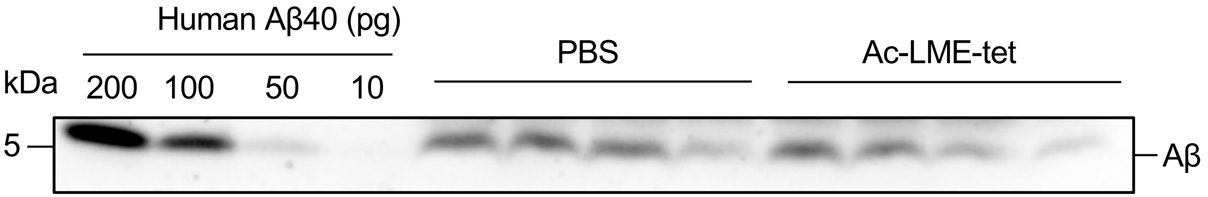

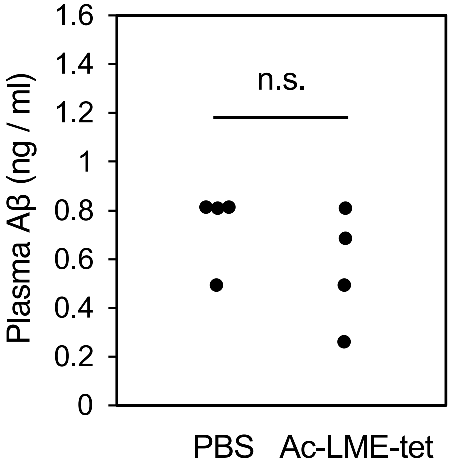

Supplementary Fig. 11: Ac-LME-tet does not affect the amount of Aβ in the plasma of AD model mice.

App$^{NL\text{-}G\text{-}F/NL\text{-}G\text{-}F}$ mice were intraperitoneally administered Ac-LME-tet or PBS control. After 7 days, the mice were sacrificed, and the amount of human Aβ present in the plasma was measured by western blotting using the anti-human Aβ antibody 4G8. The indicated amounts of Aβ were used for quantification; $n = 4$. Significance was determined using the Mann-Whitney *U* test; n.s., not significant.

Supplementary Fig. 12

a

peptide #4 MHLVICDCYCTTDICYCYSCTPN

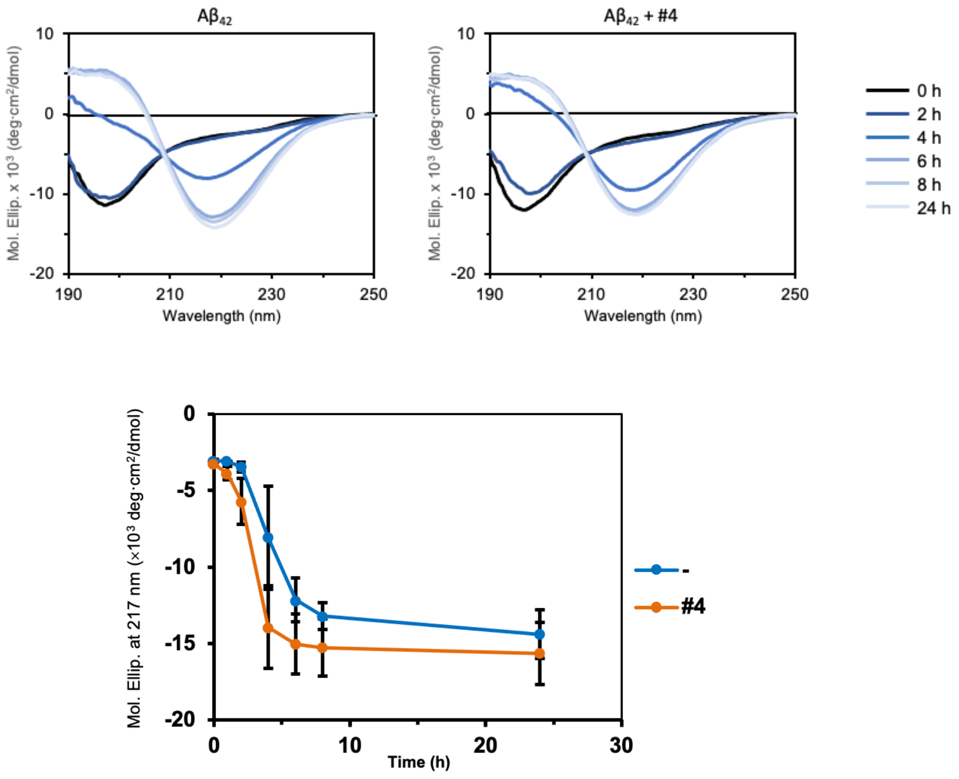

b

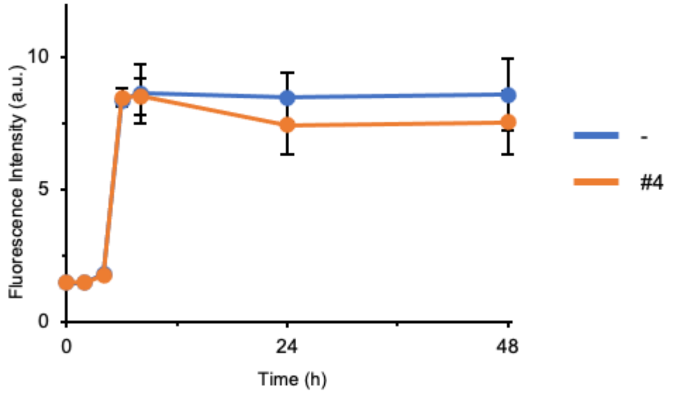

Supplementary Fig. 12: Peptide #4 does not affect the conformational change and subsequent fibrillation of Aβ42.

a Time-dependent conformational change in Aβ42 (20 μM) in the presence or absence of peptide #4 (2 μM). Far-UV CD spectra (wavelength 190-250 nm) were measured at the indicated time periods. Kinetic rates of β-sheet formation of Aβ42 were estimated by measuring time-dependent alterations of molar ellipticities of CD spectra at 217 nm. Data represent mean ± SE, $n = 3$. **b** Fibrillation profiles of Aβ42 in the presence or absence of peptide #4 (2 μM), measured by the Thioflavin T-binding assay. Time-dependent increments of Aβ42-Thioflavin T binding are shown. Data represent mean ± SE, $n = 3$.
