# Supplementary material for: A tailored tetravalent peptide displays dual functions to inhibit amyloid β production and aggregation
Source: Commun Biol. 2023 Apr 8;6:383. doi: 10.1038/s42003-023-04771-9 (PMC10082830; doi:10.1038/s42003-023-04771-9)
Supplement: Supplementary file 3 — Description of Additional Supplementary Files [file 42003_2023_4771_MOESM3_ESM.pdf]

Description of Additional Supplementary Files

File name: Supplementary Data 1

Description: The source data for Figs. 2b-c, 3a-b, 3d, 4a-d, 5a, 5d, and 6 and Supplementary Figs. 2, 8, and 11 are provided.
