# Supplementary material for: A tailored tetravalent peptide displays dual functions to inhibit amyloid β production and aggregation
Source: Commun Biol. 2023 Apr 8;6:383. doi: 10.1038/s42003-023-04771-9 (PMC10082830; doi:10.1038/s42003-023-04771-9)
Supplement: Supplementary file 5 — Reporting Summary [file 42003_2023_4771_MOESM5_ESM.pdf]

nature portfolio

Corresponding author(s): Kiyotaka Nishikawa, Satoru Funamoto

Last updated by author(s): Mar 13, 2023

Reporting Summary

Nature Portfolio wishes to improve the reproducibility of the work that we publish. This form provides structure for consistency and transparency in reporting. For further information on Nature Portfolio policies, see our Editorial Policies and the Editorial Policy Checklist.

Statistics

For all statistical analyses, confirm that the following items are present in the figure legend, table legend, main text, or Methods section.

n/a	Confirmed	
☐	☒	The exact sample size (*n*) for each experimental group/condition, given as a discrete number and unit of measurement
☐	☒	A statement on whether measurements were taken from distinct samples or whether the same sample was measured repeatedly
☐	☒	The statistical test(s) used AND whether they are one- or two-sided<br>*Only common tests should be described solely by name; describe more complex techniques in the Methods section.*
☒	☐	A description of all covariates tested
☐	☒	A description of any assumptions or corrections, such as tests of normality and adjustment for multiple comparisons
☐	☒	A full description of the statistical parameters including central tendency (e.g. means) or other basic estimates (e.g. regression coefficient) AND variation (e.g. standard deviation) or associated estimates of uncertainty (e.g. confidence intervals)
☐	☒	For null hypothesis testing, the test statistic (e.g. *F*, *t*, *r*) with confidence intervals, effect sizes, degrees of freedom and *P* value noted<br>*Give P values as exact values whenever suitable.*
☒	☐	For Bayesian analysis, information on the choice of priors and Markov chain Monte Carlo settings
☒	☐	For hierarchical and complex designs, identification of the appropriate level for tests and full reporting of outcomes
☒	☐	Estimates of effect sizes (e.g. Cohen's *d*, Pearson's *r*), indicating how they were calculated

Our web collection on statistics for biologists contains articles on many of the points above.

Software and code

Policy information about availability of computer code

Data collection	ZEN (ZEISS)
Data analysis	Statistical analysis: IBM SPSS Statistics software (ver. 26.0.0.0).

For manuscripts utilizing custom algorithms or software that are central to the research but not yet described in published literature, software must be made available to editors and reviewers. We strongly encourage code deposition in a community repository (e.g. GitHub). See the Nature Portfolio guidelines for submitting code & software for further information.

Data

Policy information about availability of data

All manuscripts must include a data availability statement. This statement should provide the following information, where applicable:

- Accession codes, unique identifiers, or web links for publicly available datasets
- A description of any restrictions on data availability
- For clinical datasets or third party data, please ensure that the statement adheres to our policy

All data are available from corresponding authors upon reasonable request.

Human research participants

Policy information about studies involving human research participants and Sex and Gender in Research.

Reporting on sex and gender	N/A
Population characteristics	N/A
Recruitment	N/A
Ethics oversight	N/A

Note that full information on the approval of the study protocol must also be provided in the manuscript.

Field-specific reporting

Please select the one below that is the best fit for your research. If you are not sure, read the appropriate sections before making your selection.

☒ Life sciences ☐ Behavioural & social sciences ☐ Ecological, evolutionary & environmental sciences

For a reference copy of the document with all sections, see nature.com/documents/nr-reporting-summary-flat.pdf

Life sciences study design

All studies must disclose on these points even when the disclosure is negative.

Sample size	No statistical methods were used to determine the sample size. We repeated each experiment at least three times and results were reproducibly obtained.
Data exclusions	No data were excluded.
Replication	All attempts at replication were successful.
Randomization	N/A
Blinding	N/A

Reporting for specific materials, systems and methods

We require information from authors about some types of materials, experimental systems and methods used in many studies. Here, indicate whether each material, system or method listed is relevant to your study. If you are not sure if a list item applies to your research, read the appropriate section before selecting a response.

Materials & experimental systems

n/a	Involved in the study	
☐	☒	Antibodies
☐	☒	Eukaryotic cell lines
☒	☐	Palaeontology and archaeology
☐	☒	Animals and other organisms
☒	☐	Clinical data
☒	☐	Dual use research of concern

Methods

n/a	Involved in the study	
☒	☐	ChIP-seq
☒	☐	Flow cytometry
☒	☐	MRI-based neuroimaging

Antibodies

Antibodies used	mouse anti-human APP antibody 6E10 (Biolegend, San Diego, CA, USA, Cat# 803001), mouse anti-human Aβ antibody 82E1 (Immuno-Biological Laboratories Co., Ltd., Gumma, Japan, Cat# 10323),rabbit anti-human Aβ42 specific antibody (Immuno-Biological Laboratories Co., Ltd., Cat#18582), mouse anti-Aβ17-24 antibody 4G8 (Biolegend, Cat#800709), rabbit anti-sAPPβ antibody (Immuno-Biological Laboratories Co., Ltd., Cat# 18957), rabbit anti-EEA1 antibody (Thermo Fisher Scientific, Waltham, MA, USA, Cat# PA1-063A),rabbit polyclonal anti-LAMP1 antibody (Abcam, Cambridge, UK, Cat#ab24170),rabbit polyclonal anti-SQSTM1/p62 antibody (MBL International, Woburn, MA, USA, Cat#PM045), rabbit polyclonal anti-LC3 antibody (MBL International, Cat#PM036), rabbit polyclonal anti-β-actin antibody (MBL International, Cat# PM-053),rabbit anti-Sialyltransferase 1 (St6gal1) antibody (Immuno-

Biological Laboratories Co., Ltd., Cat# 18983),mouse anti-Myc-Tag antibody 9B11 (Cell Signaling Technology, Danvers, MA, USA, Cat# 2276), horseradish peroxidase (HRP)-conjugated goat anti-rabbit IgG antibody (Cell Signaling Technology, Cat# 7074S),HRP-conjugated horse anti-mouse IgG antibody (Cell Signaling Technology, Cat# 7076S), Alexa Fluor 488-conjugated goat anti-mouse IgG antibody(Thermo Fisher Scientific, Cat# A-11001), Alexa Fluor 546-conjugated goat anti-rabbit IgG antibody(Thermo Fisher Scientific, Cat# A-11010), Alexa Fluor 546-conjugated goat anti-mouse IgG antibody (Thermo Fisher Scientific, Cat# A-11003).

Validation	All purchased antibodies were well validated by the manufactures in their specific data sheets or other researchers in the previous literatures.Prior to using, we tested the reactivity of the purchased antibodies against the samples alongside the positive control ensured in the manufacture's data sheet.

Eukaryotic cell lines

Policy information about cell lines and Sex and Gender in Research

Cell line source(s)	Chinese Hamster Ovary (CHO) cells stably expressing human APP (APP1-751) was kindly provided by Dr. E.H. Koo, University of California, San Diego. 7WD10 cells stably expressing Myc-tagged Notch with a deletion in the extracellular region (NotchΔE) and 7WD10-NotchΔE cells stably expressing β-galactoside alpha-2,6-sialyltransferase 1 (St6gal1; 7WD10-Notch ΔE-St6gal1 cells) were generated as described previously (Funamoto S. et.al., Nat Commun.,2013, doi: 10.1038/ncomms3529).
Authentication	All CHO cells used in this study were negative for the contamination of at least human derived cells, as tested by previously established procedure (Cytotechnology, 2009, doi: 10.1007/s10616-009-9245-5).
Mycoplasma contamination	All the cell lines were negative for Mycoplasma contamination test.
Commonly misidentified lines (See ICLAC register)	No commonly misidentified cell lines were used.

Animals and other research organisms

Policy information about studies involving animals; ARRIVE guidelines recommended for reporting animal research, and Sex and Gender in Research

Laboratory animals	7-week-old mice with single humanized App KI, carrying Swedish (NL), Beyreuther/Iberian (F), and Arctic (G) mutations (AppNL-G-F/NL-G-F mice)
Wild animals	This study didn't involve wild animals.
Reporting on sex	Both male and female mice were used in this study.
Field-collected samples	This study didn't involve samples collected from the field.
Ethics oversight	All animal experiments were approved by the Animal Ethics Committee of Doshisha University prior to their commencement and performed in accordance with approved protocols.

Note that full information on the approval of the study protocol must also be provided in the manuscript.
